# Comparison of natural language processing algorithms in assessing the importance of head computed tomography reports written in Japanese

**DOI:** 10.1007/s11604-024-01549-9

**Published:** 2024-03-29

**Authors:** Tomohiro Wataya, Azusa Miura, Takahisa Sakisuka, Masahiro Fujiwara, Hisashi Tanaka, Yu Hiraoka, Junya Sato, Miyuki Tomiyama, Daiki Nishigaki, Kosuke Kita, Yuki Suzuki, Shoji Kido, Noriyuki Tomiyama

**Affiliations:** 1 Department of Radiology, Osaka University Graduate School of Medicine, 2-2, Yamadaoka, Suita, Osaka 565-0871 Japan; 2 Department of Artificial Intelligence Diagnostic Radiology, Osaka University Graduate School of Medicine, 2-2, Yamadaoka, Suita, Osaka 565-0871 Japan; 3 Department of Diagnostic Imaging, Osaka General Medical Center, 3-1-56. Mandai Higashi, Sumiyoshi, Osaka 558-8558 Japan; 4 Department of Diagnostic Radiology, Sakai City Medical Center, 1-1-1, Ebaracho, Sakai, Osaka 593-8304 Japan; 5 Division of Health Science, Osaka University Graduate School of Medicine, 1-7, Yamadaoka, Suita, Osaka 565-0871 Japan

**Keywords:** Artificial Intelligence, Natural Language Processing, Radiology Report, Report Importance, Risk Management

## Abstract

**Purpose:**

To propose a five-point scale for radiology report importance called *Report Importance Category* (RIC) and to compare the performance of natural language processing (NLP) algorithms in assessing RIC using head computed tomography (CT) reports written in Japanese.

**Materials and methods:**

3728 Japanese head CT reports performed at Osaka University Hospital in 2020 were included. RIC (category 0: no findings, category 1: minor findings, category 2: routine follow-up, category 3: careful follow-up, and category 4: examination or therapy) was established based not only on patient severity but also on the novelty of the information. The manual assessment of RIC for the reports was performed under the consensus of two out of four neuroradiologists. The performance of four NLP models for classifying RIC was compared using fivefold cross-validation: logistic regression, bidirectional long–short-term memory (BiLSTM), general bidirectional encoder representations of transformers (general BERT), and domain-specific BERT (BERT for medical domain).

**Results:**

The proportion of each RIC in the whole data set was 15.0%, 26.7%, 44.2%, 7.7%, and 6.4%, respectively. Domain-specific BERT showed the highest accuracy (0.8434 ± 0.0063) in assessing RIC and significantly higher AUC in categories 1 (0.9813 ± 0.0011), 2 (0.9492 ± 0.0045), 3 (0.9637 ± 0.0050), and 4 (0.9548 ± 0.0074) than the other models (*p* < .05). Analysis using layer-integrated gradients showed that the domain-specific BERT model could detect important words, such as disease names in reports.

**Conclusions:**

Domain-specific BERT has superiority over the other models in assessing our newly proposed criteria called RIC of head CT radiology reports. The accumulation of similar and further studies of has a potential to contribute to medical safety by preventing missed important findings by clinicians.

**Supplementary Information:**

The online version contains supplementary material available at 10.1007/s11604-024-01549-9.

## Introduction

Because radiology reports contain information about various abnormalities or findings, incidental findings such as lung masses found on post-traumatic computed tomography (CT) scans [1] can be described. However, it has been reported that up to 8.0% of clinicians often do not review reports [2], which is a major medical safety concern, because missed findings can lead to delays in therapeutic intervention and ultimately to patient disability or death. Despite the development of systems that allow radiologists to alert clinicians to important or actionable findings [3], this is imperfect, because the standards for alerting are left to the subjectivity of the radiologist. Thus, objective standards for the importance of radiology reports and, if possible, systems that allow for automatic assessment of importance are needed.

Because radiology reports are often described in free-text format, they have attracted attention as challenging targets for natural language processing (NLP). Such trends are strongly supported by advances in NLP technology. One of the traditional NLP approaches was the rule-based algorithm, in which texts are classified according to rules established by human experts, such as whether they contain certain words. Then, statistical NLP models such as count-based algorithms emerged, where text features are calculated by the counts of word frequencies [4]. With the development of deep learning, long–short-term memory (LSTM) [5] was proposed. Before the advent of LSTM, deep learning models, such as convolutional neural network, could only process data of a fixed size and were not suited to processing data of an indefinite length, such as free text. LSTM has overcome this barrier by adopting recurrent neural networks capable of sequential word-by-word processing. Researches using LSTM on radiology reports have already been performed, such as detecting bone metastasis [6]. However, LSTM only allowed words to be entered in the order in which they appeared in a sentence (or vice versa), which has been an obstacle to NLP, where the engagement of words that are apparently far apart is important. Recently, bidirectional encoder representations from transformers (BERT) [7] have been proposed, which were equipped with multiple self-attention layers. In self-attention layers, the strength of the connection between words is inferred by calculating the attention weights. BERT models have outperformed previous models in NLP tasks, such as document classification and question answering [7]. BERT models are often pre-trained with a large amount of text data, and models are available not only for English but also for other languages, such as Japanese [8]. In addition to BERT models pre-trained with general language data, there are also models pre-trained with domain-specific data, such as medical data [9]. Previous studies to assess the importance of reports have demonstrated the usefulness of NLP, such as characterizing changes and significance of clinical findings using a count-based model [10] and classifying reports with or without actionable alerts by radiologists using BERT [11]. We believe that to clinically implement systems for grading the importance of radiology reports, NLP models should be trained using reports annotated according to objective criteria, but there are few such studies, especially after the advent of BERT.

We believe that NLP can be a key for assessing the importance of radiology reports written in Japanese. In this research, to obtain a more accurate NLP model, we compared four NLP schemes: count-based, LSTM, general BERT, and domain-specific BERT models. However, as far as we know, there are no generally accepted scales of report importance, so we initially defined the scale called *Report Importance Category* (RIC), which is a five-point scale and is based not only on patient severity but also on the novelty of information in the reports. Thus, the proposal of RIC is another purpose of this research. Since there are many imaging modalities and target body parts, we decided to focus on plain head CT reports to limit the variation in findings. If successful, systems can be developed to urge radiologists to issue alerts to physicians when reports with critical findings are generated, which can contribute to improved medical safety. As such, this study has significant potential to revolutionize current practice and pave the way for the development of new, automated report-scoring systems.

## Materials and methods

Approval for this study was obtained from the internal Ethics Review Board of Osaka University Hospital (Suita, Japan). The need for informed consent was waived because of the retrospective nature of this study.

### Study population and data preprocessing

**Fig. 1:**
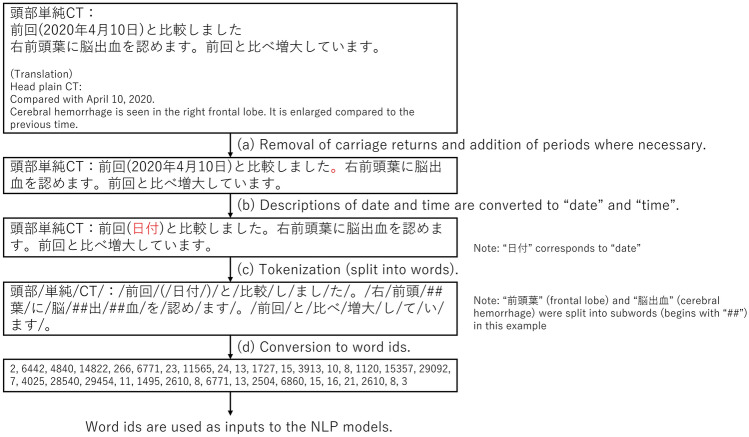
Overview of preprocessing and tokenization. The report (fictitious for illustrative purposes) is preprocessed and tokenized. The result of the tokenization is by the tokenizer used in general BERT. *NLP* natural language processing

In 2020, reports for 3738 plain head CT examinations were issued at Osaka University Hospital, and the reports were included. Ten reports were excluded, because they did not include descriptions of the brain or skull. As a result, 3728 reports were included. In this study, only the findings (main text) of the reports were included, not the diagnoses (conclusions) or the order information. Since BERT cannot recognize carriage returns, they were excluded from the report, and periods were inserted where necessary (when the previous sentence lacked the period) (Fig. 1a). In addition, to minimize the variation caused by the date and time of the examination, they were converted to “date” and “time” (described in Japanese), respectively, using regular expressions (Fig. 1b).

### Report importance category (RIC)

The definitions for the importance of the report were established in five categories based on the importance of the findings themselves and their clinical course; category 0: no findings, category 1: minor findings, category 2: routine follow-up, category 3: careful follow-up, and category 4: examination or therapy. A detailed explanation of each category is given in Table 1. To take an example of “cerebral hemorrhage”, category 1: “old hemorrhage”, category 2: “existing hemorrhage reduces or does not change”, category 3: “existing hemorrhage expands”, while category 4: “new hemorrhage” or “existing hemorrhage expands to the extent that it complicates brain herniation”.

**Table 1:** Summary of RIC

RIC	Meaning	Explanation
Category 0	No findings	No findings are described
Category 1	Minor findings	Findings are described, but they do not require follow-up
Category 2	Routine follow-up	Findings are described and require "routine follow-up," meaning that clinically scheduled follow-up intervals do not need to be shortened because of the findings
Category 3	Careful follow-up	Findings are described and require "careful follow-up," meaning that the follow-up intervals for the findings should be shortened
Category 4	Examination or therapy	Novel or urgent findings are described, and further examination or therapy should be considered

*RIC* report importance category

**Fig. 2:**
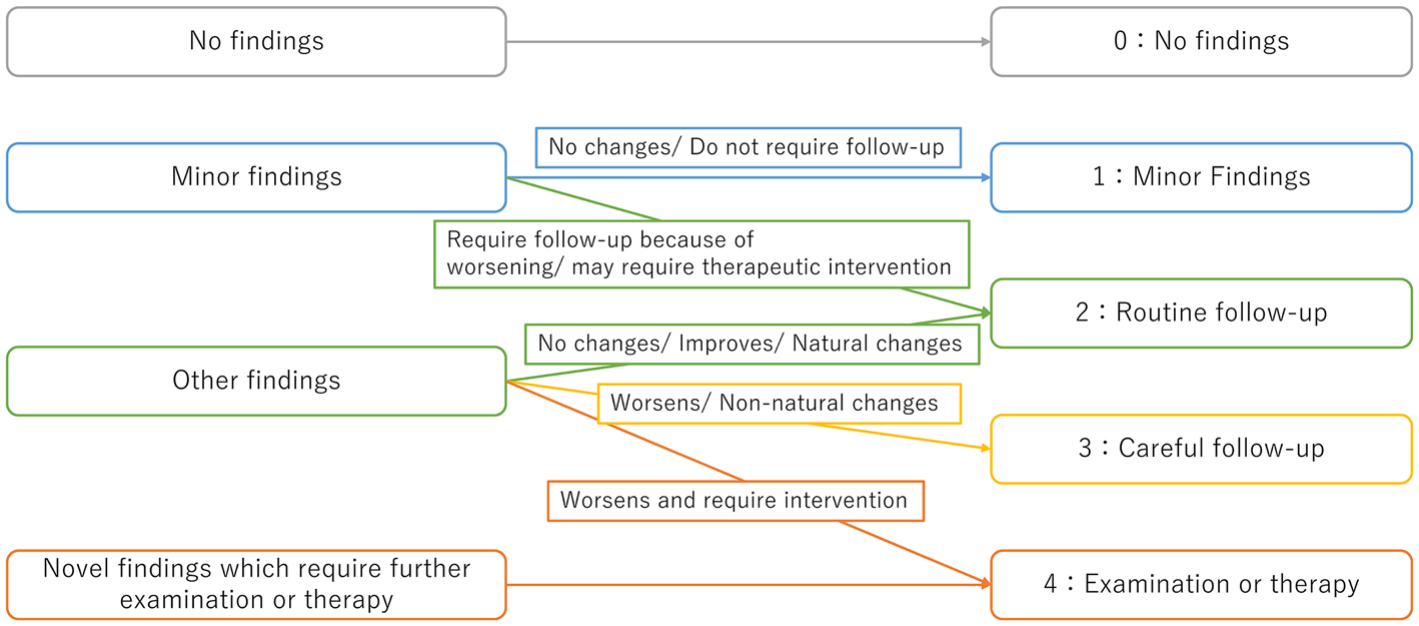
Flowchart of RIC decision. *RIC* report importance category

Here, “findings” refer to all abnormalities, including normal variants and age-related changes. When more than one finding is described in a report, we take the one with the highest category. Figure 2 shows the categorization flowchart, and detailed definitions are provided in Supplementary Material 1.

Manual annotation of RIC for the 3728 reports was performed by four radiologists specializing in neuroradiology with 4, 4, 4, and 6 years of clinical experience. They received a detailed lecture on RIC. They shared the assignment, so that for each report, two out of the four radiologists graded RIC independently. If agreement was reached, the assessment was accepted (“agreed reports”), if not, another senior neuroradiologist with 29 years of clinical experience selected one of the assessments (“disagreed reports”).

### Data split

**Fig. 3:**
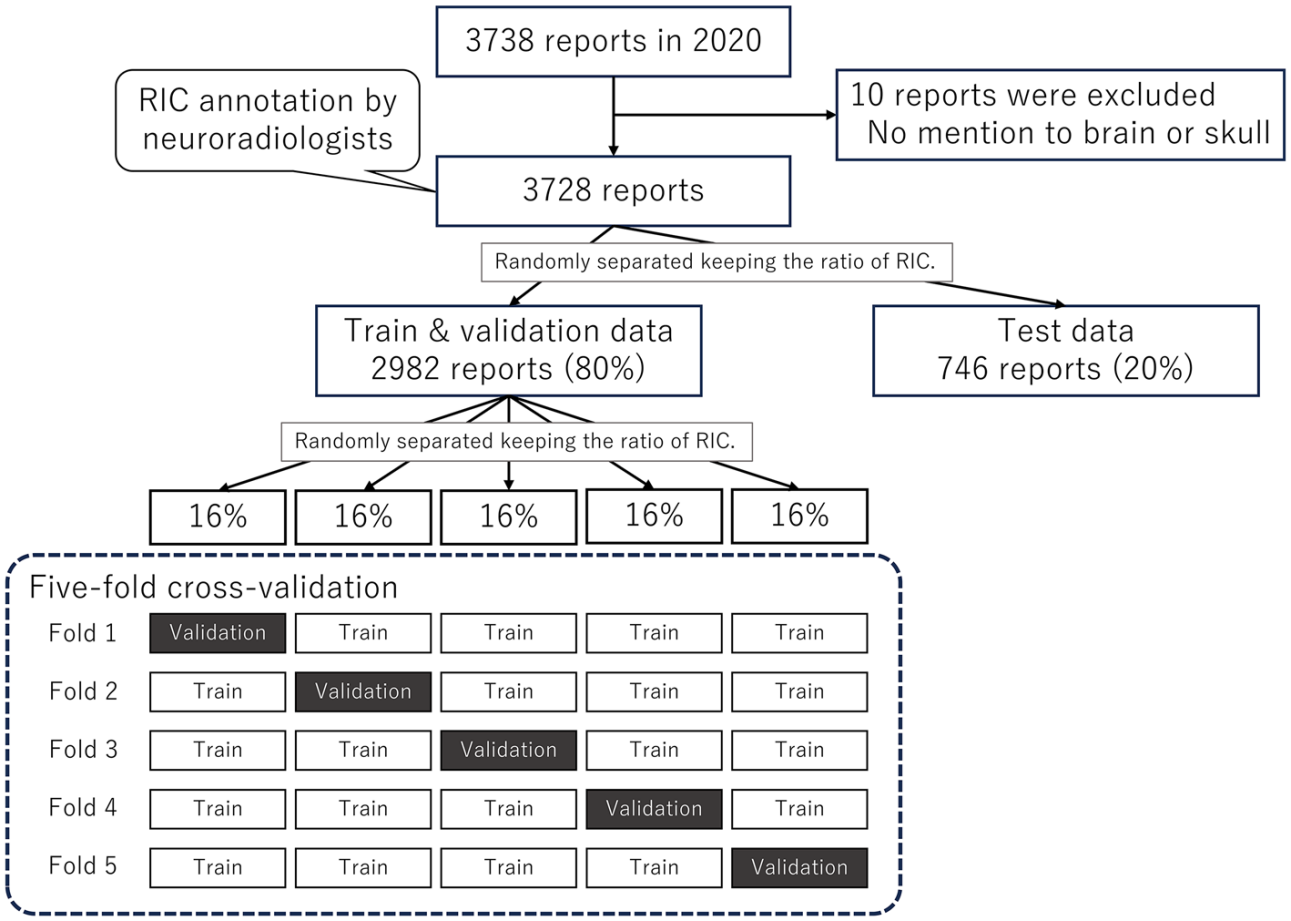
Overview of data split. *RIC* report importance category

The annotated 3728 reports were randomly, but keeping the ratio of *RIC*, separated into the train & validation data (80%: 2982 reports) and the test data (20%: 746 reports). In this research, fivefold cross-validation was conducted, where the train and validation data were separated into five groups and in each fold, the models were trained using four groups and validated by one group, and the group used for validation was changed. The overview of the data split is shown in Fig. 3.

### Tokenization

To input sentences, they must be segmented into tokens (words). Since Japanese sentences are not separated by spaces like such languages as English, tokenizing algorithms developed for Japanese are different from those used for those other languages. In BERT models targeted on Japanese [8, 9], texts are first separated using morphological analysis using such analyzers as MeCab [12] (Fig. 1c). MeCab can refer to external dictionaries, such as mecab-ipadic-NEologd dictionary, which contains commonly used words including proper nouns [13], and J-MeDic, which is composed of medical terms [14]. Morphological analysis is followed by Wordpiece [15] tokenization, in which some words, especially those rare in pretraining corpus, are separated into subwords, so that the number of token types becomes the designated number (a hyperparameter in pretrain). Subwords are often distinguished by starting with a double hash (##subword). As described later, two BERT models were used in this study: general BERT and domain-specific BERT. The former [8] uses the mecab-ipadic-NEologd dictionary as an external dictionary and has 32,768 tokens, whereas the latter [9] model uses the mecab-ipadic-NEologd dictionary and J-MeDic and has 25,000 tokens. Tokens obtained from the reports are converted to numbers (word ids) according to the vocabulary defined by the tokenizer (Fig. 1d).

In models other than BERT, there is no fixed tokenizer. Although methods such as Sentencepiece [16], which extracts tokens using the entire corpus without using external dictionaries, are commonly used, in this study, the one for domain-specific BERT was used to minimize the differences caused by differences in tokenizers. However, words that occur less than 10 times in the train and validation data were replaced with unknown tokens ([UNK]) to avoid insufficient training on scarce words.

### NLP models and study settings

**Fig. 4:**
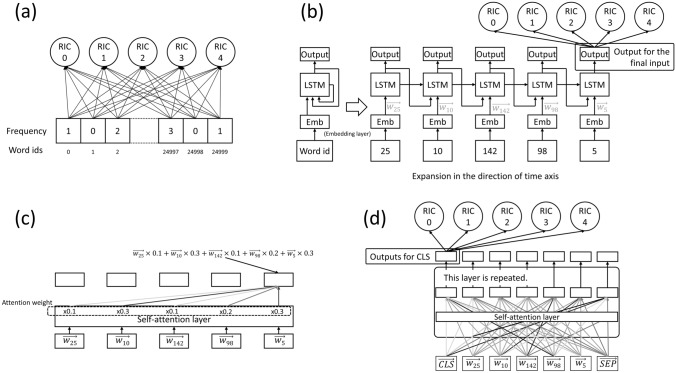
Diagrams of NLP models. **a** Count-based model, **b** LSTM model (for simplicity, this figure is for the single directional LSTM. The actual BiLSTM model is composed of two such structures: one handles sentences from the beginning to the end, whereas the other vice versa.), **c** self-attention layer, and **d** BERT model. *NLP* natural language processing. *RIC* report importance category, *LSTM* long–short-term memory, *BERT* bidirectional encoder representations from transformers

This experiment was conducted using Python 3.8 and PyTorch 1.10.1. Details of the models used in this experiment are as follows:

(a)* Count-based model (logistic regression)* [4]: Each report is converted into a 25,000-dimensional feature vector based on the frequency of the tokens (words) that make it up. Feature vectors were used for RIC induction with logistic regression. In this study, we did not adopt the regularization technique. Figure 4a shows a schematic diagram of this technique. Each arrow is assigned a weight, which is the trainable parameter.

(b)* Bidirectional LSTM (BiLSTM)* [17]: LSTM is a type of recurrent neural network that takes one token at a time as input and produces an output corresponding to the input. When processing word ids, they are converted to vectors using the embedding layer before input to the LSTM layer. In the LSTM layer, the data stored in the model is considered along with the output for the previous token, allowing the model to understand word-to-word ordering [5]. Figure 4b shows the diagram of LSTM (for simplicity, this figure is for the single directional LSTM). BiLSTM is a type of LSTM that can process tokens from the beginning of a sentence to the end and vice versa. The output for the last input was used for RIC induction using a fully connected layer.

*BERT models* [7]: BERT models are composed of multiple self-attention layers and can handle relations between distant words in the documents. Figure 4c shows the diagram of self-attention layer. Strength of the connection between tokens are called attention weights and calculated through this layer. BERT models are composed of the repetition of self-attention layers (Fig. 4d). Commonly, the CLS token is given at the beginning of the sentence and the SEP token at the end, and the output for the CLS token is considered the output for the entire sentence. Models are often pre-trained with a large amount of text and fine-tuned in experiments. In this research, two pre-trained Japanese BERT models were used:

(c)* General BERT* [8]: This BERT was pre-trained with the Japanese Wikipedia corpus, which consists of non-medical sentences.

(d)* Domain-specific BERT* (UTH-BERT [9]): This BERT model was pre-trained with Japanese medical records from Tokyo University Hospital.

The hyperparameters for the DL-based models (LSTM and BERT) were determined using Optuna, the auto-optimization framework for hyperparameters [18]. The hyperparameters used in this experiment are listed in Supplementary Material 2.

Since fivefold cross-validation was performed in this research, trained model with different parameters was produced through each fold. The output probabilities for each RIC for the test data derived from five models were averaged to calculate the final outputs of the NLP algorithm.

### Statistical analysis

For each report, the NLP models output the possibilities for each of the five categories. Since fivefold cross-validation was used in this research, five models with different weights were produced for each NLP method. The accuracy, F1 scores (the macro-F1 score and F1 score for each category), areas under the receiver operating characteristic (ROC) curves (AUCs: the macro-AUC and AUC score for each category) for the test data were calculated using Scikit-learn 1.2.1 [19] for each fold. These were compared between the NLP methods by the Mann–Whitney *U* test using SciPy 1.10.0 [20], and *p* values < 0.05 were considered significant.

In addition, to intuitively visualize the differences among models, ROC curves and confusion metrics for the test data set were drawn. When drawing, the outputs (possibility for each category) derived from each fold model were averaged (these values were not used for statistical analysis).

### Interpretation of reasons for categorization

In logistic regression, the weight for each category is calculated for each token. For each category, sorting the tokens by weight can reveal the tokens that characterize the category. In this research, tokens were listed for the top five weights for each category.

To interpret the reasons for categorization by BiLSTM and BERT models layer integrated gradients (LIG) [21] were performed to highlight the part of the reports that the model focuses on. LIG calculates what part of the output of a particular layer in the model contributed to the final output of the model by integrating the gradient. In this research, we applied LIG to the outputs of the embedding layer, where the results (LIG scores) can be interpreted as outputs for each token. The LIG scores were divided by the norm of the scores of the whole report and visualized in 256 shades of red, which means that the stronger the red color is, the more the token contributes to the output of the model.

## Results

### Data set

During the manual annotation process, 532 out of 3728 reports (14.3%) were disagreed reports and were re-annotated by the senior neuroradiologist. Of these, 109 were included in the test data set. Table 2 shows the characteristics of the entire data set. Category 2 reports accounted for the largest number of reports (44.2%), while reports with severe content (category 4) were less common (6.4%). The number of tokens per report tended to increase with higher categories (i.e., reports tended to be longer).

**Table 2:** Breakdown of categories and number of tokens in the data set

	Number of reports (%)	Tokens per report (average ± SD)
Category 0	561(15.0)	19.95 ± 8.88
Category 1	995(26.7)	47.41 ± 20.59
Category 2	1646(44.2)	69.46 ± 27.25
Category 3	288(7.7)	87.37 ± 34.76
Category 4	238(6.4)	83.03 ± 37.51
Total	3728	58.37 ± 32.38

*SD* standard deviation

### Performance of categorization of models

Table 3 shows the accuracy and macro-F1 score of each model for the test data set. Domain-specific BERT performed best with the accuracy of 0.8434 ± 0.0063, significantly higher than logistic regression (0.7871 ± 0.0066) and BiLSTM (0.7654 ± 0.0086), whereas higher but not significant than general BERT (0.8164 ± 0.0161). In the domain-specific BERT, the macro-F1 score (0.7826 ± 0.0242) and macro-AUC (0.9693 ± 0.0032) were significantly higher than the other models. In domain-specific BERT, F1 scores and AUCs for categories 3 and 4 were lower than those for categories 0–2 for all models. Supplemental material 3 shows the performance of the model derived from each fold for train, validation and test data sets, showing almost all scores tended to decrease in the validation and test data sets compared to the train data set.

**Table 3:** Accuracies, macro-F1 scores and AUCs of each NLP models

	Logistic regression (LR)	BiLSTM (BL)	General BERT (GB)	Domain-specific BERT (DB)	*p* values					
					LR vs BL	LR vs GB	LR vs DB	BL vs GB	BL vs DB	GB vs DB
All reports										
Accuracy	0.7871 ± 0.0066	0.7654 ± 0.0086	0.8164 ± 0.0161	0.8434 ± 0.0063	0.0079*	0.0159*	0.0079*	0.0079*	0.0079*	0.0556
Macro-F1	0.6942 ± 0.0147	0.5963 ± 0.0319	0.7287 ± 0.0264	0.7826 ± 0.0242	0.0079*	0.0952	0.0079*	0.0079*	0.0079*	0.0317*
F1 for category 0	0.9434 ± 0.0040	0.9570 ± 0.0093	0.9782 ± 0.0035	0.9801 ± 0.0036	0.0937	0.0119*	0.0119*	0.0079*	0.0079*	0.5982
F1 for category 1	0.8254 ± 0.0095	0.8278 ± 0.0116	0.8555 ± 0.0126	0.8764 ± 0.0075	0.6905	0.0159*	0.0079*	0.0159*	0.0079*	0.0556
F1 for category 2	0.8080 ± 0.0062	0.7868 ± 0.0046	0.8291 ± 0.0159	0.8507 ± 0.0047	0.0079*	0.0465*	0.0079*	0.0079*	0.0079*	0.0952
F1 for category 3	0.4886 ± 0.0370	0.1067 ± 0.1097	0.5428 ± 0.0898	0.6111 ± 0.0822	0.0079*	0.2222	0.0952	0.0079*	0.0079*	0.0952
F1 for category 4	0.4053 ± 0.0519	0.3035 ± 0.0588	0.4379 ± 0.0555	0.5950 ± 0.0427	0.0556	0.4206	0.0079*	0.0079*	0.0079*	0.0079*
Macro-AUC	0.9203 ± 0.0055	0.9100 ± 0.0077	0.9512 ± 0.0064	0.9693 ± 0.0032	0.0952	0.0079*	0.0079*	0.0079*	0.0079*	0.0079*
AUC for category 0	0.9957 ± 0.0007	0.9907 ± 0.0024	0.9960 ± 0.0031	0.9974 ± 0.0014	0.0079*	1.0000	0.0361*	0.0317*	0.0119*	0.6752
AUC for category 1	0.9528 ± 0.0008	0.9570 ± 0.0041	0.9711 ± 0.0042	0.9813 ± 0.0011	0.1425	0.0119*	0.0119*	0.0079*	0.0079*	0.0079*
AUC for category 2	0.9032 ± 0.0047	0.8853 ± 0.0100	0.9303 ± 0.0079	0.9492 ± 0.0045	0.0317*	0.0079*	0.0079*	0.0079*	0.0079*	0.0159*
AUC for category 3	0.8917 ± 0.0115	0.8492 ± 0.0188	0.9402 ± 0.0100	0.9637 ± 0.0050	0.0159*	0.0079*	0.0079*	0.0079*	0.0079*	0.0079*
AUC for category 4	0.8580 ± 0.0174	0.8679 ± 0.0130	0.9185 ± 0.0147	0.9548 ± 0.0074	0.6905	0.0079*	0.0079*	0.0079*	0.0079*	0.0079*
Agreed reports										
Accuracy	0.8361 ± 0.0081	0.8056 ± 0.0093	0.8700 ± 0.0175	0.9030 ± 0.0098	0.0117*	0.0361*	0.0119*	0.0119*	0.0119*	0.0278*
Macro-F1	0.7409 ± 0.0204	0.6275 ± 0.0402	0.7863 ± 0.0332	0.8556 ± 0.0298	0.0079*	0.0952	0.0079*	0.0079*	0.0079*	0.0556
Disagreed reports										
Accuracy	0.5009 ± 0.0214	0.5303 ± 0.0269	0.5028 ± 0.0455	0.4954 ± 0.0296	0.1116	1.0000	0.8330	0.4005	0.2073	0.9166
Macro-F1	0.4133 ± 0.0215	0.3801 ± 0.0481	0.4381 ± 0.0416	0.4589 ± 0.0150	0.3095	0.4206	0.0079*	0.1508	0.0079*	0.3095

*NLP* natural language processing, *BiLSTM* Bi-directional long–short term memory, *BERT* bidirectional encoder representations from transformers

*p < 0.05

**Fig. 5:**
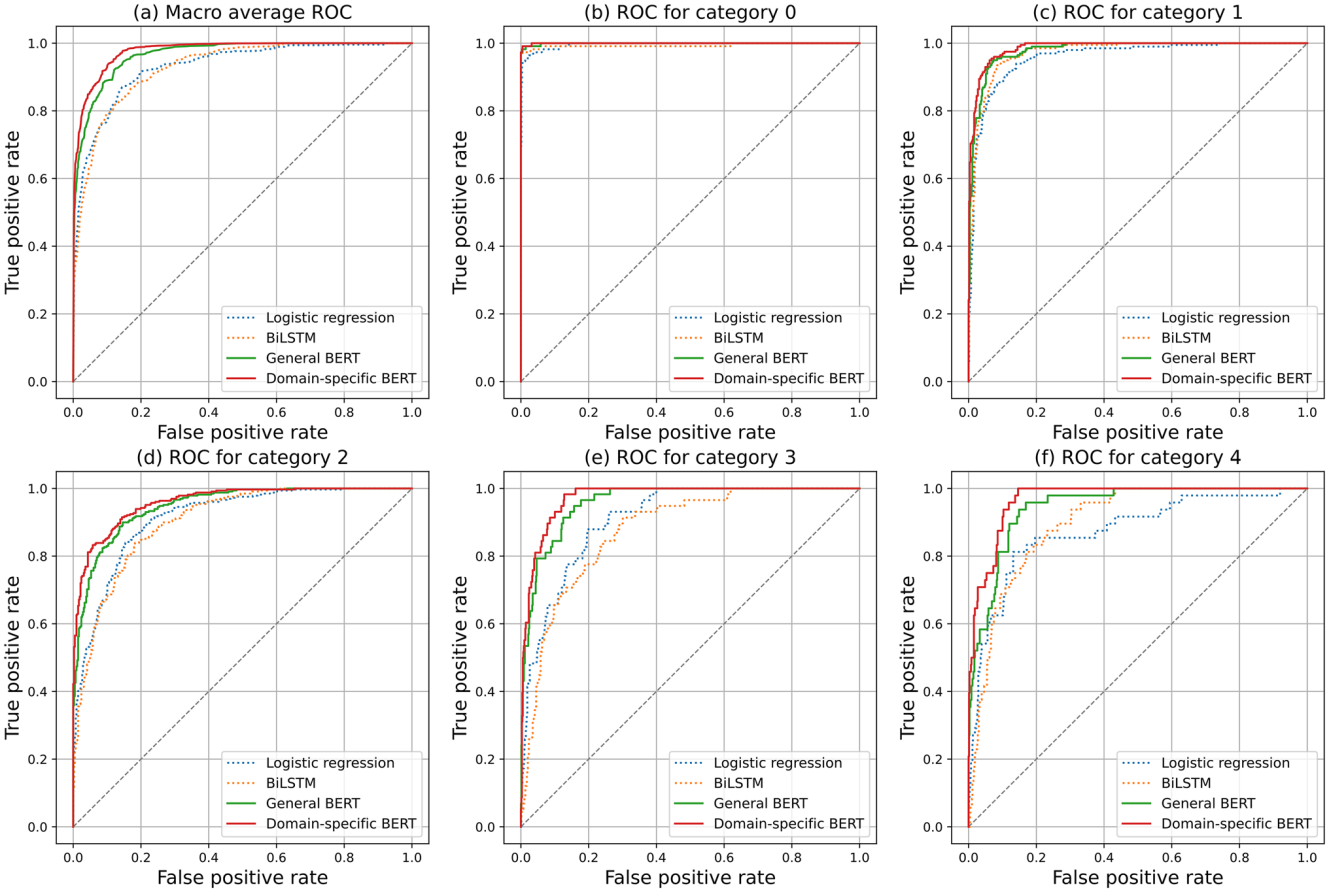
Macro-average ROC and ROC for each category. **a** shows the macro-average ROC for each NLP model. **b**–**e** show ROC for each category. *ROC* receiver operating characteristic curve, *NLP* natural language processing, *BiLSTM* Bidirectional long–short-term memory, *BERT* bidirectional encoder representations from transformers

**Fig. 6:**
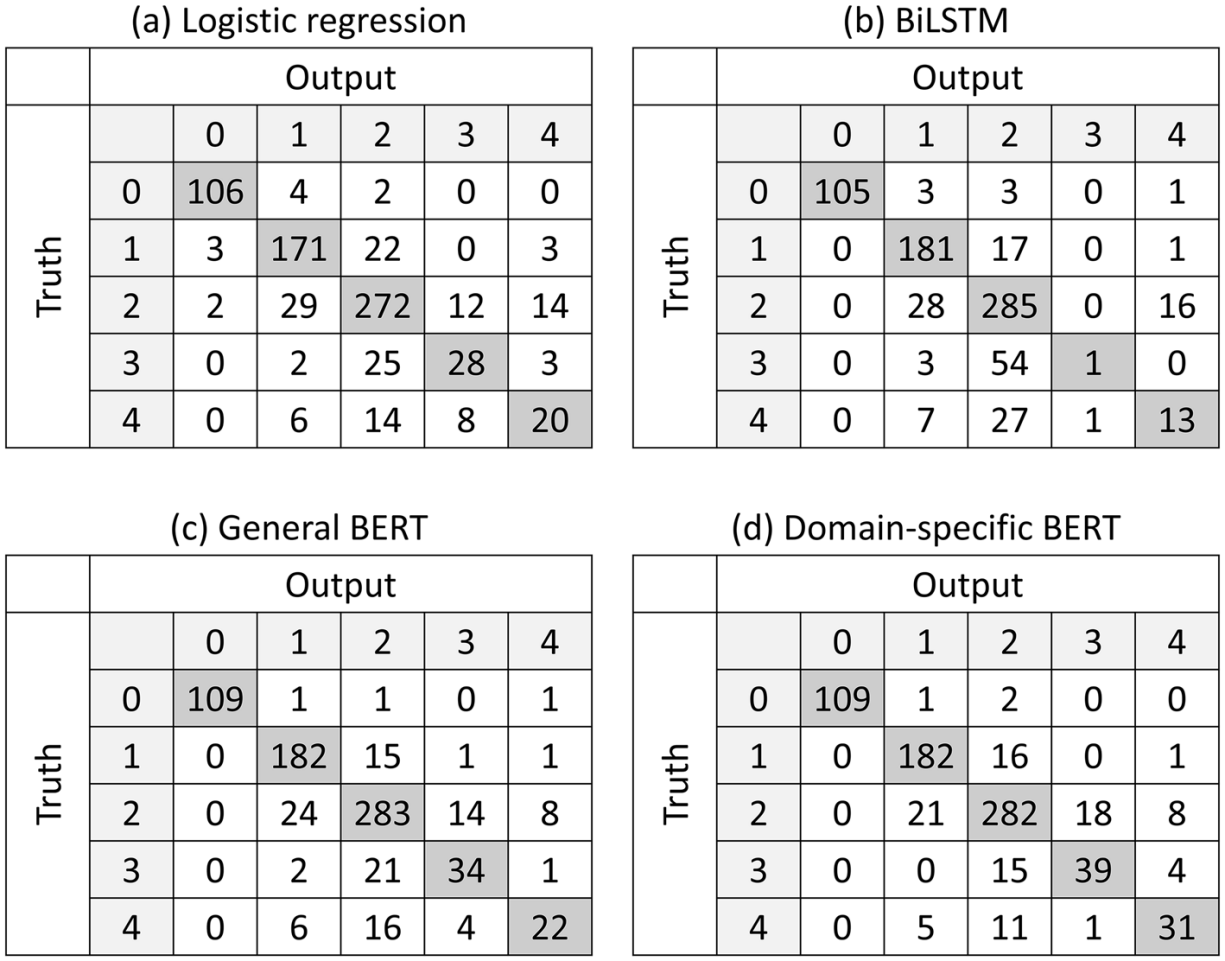
Confusion metrics for the NLP models. *NLP* natural language processing, *BiLSTM* Bi-directional long–short-term memory, *BERT* bidirectional encoder representations from transformers

Figure 5a shows the macro-average ROC curve for each model. BERT models were superior to the other models, and domain-specific BERT was even better than general BERT. Figure 5b–f shows the ROC curves for each category. Except for category 0, BERT models performed significantly better than the other models, and among BERT models, domain-specific BERT was better than general BERT. On the other hand, no significant difference was observed between logistic regression and BiLSTM. Figure 6 shows the confusion metrics for the models, showing that categories 3 and 4 reports were mostly misclassified into category 2.

Table 3 also shows the accuracy and F1 score for the agreed and disagreed reports in the manual annotation. Since in the manual annotation process, for the disagreed reports, the final decision by the senior radiologist is adopting one description from the two by the radiologists, the selected description is correct, and the other is regarded as incorrect. Thus, the average accuracy for the disagreed reports by radiologists can be regarded as 50%. In all models, the accuracy for disagreed reports was approximately 50%, which was comparable to that of the radiologists.

### Interpretation of the reasons for categorization

#### Tokens for the top five weights in logistic regression

Table 4 shows tokens for the top five weights for each category in logistic regression; the higher the weight of tokens that make up a report for a category, the more likely the report is to be classified into that category. Tokens implying minor findings such as “atrophy” and “old” (often followed by “infarction” or “hemorrhage”) are important for category 1, whereas tokens implying worsening such as “enlarge” and “expand” are important for category 3, and tokens suggesting that lesions are new such as “emergence” are important for category 4. Note that in categories 3 and 4, explicit disease names do not appear in the table, since these can also appear in category 2.

**Table 4:** Tokens for the top five weights in logistic regression for each category

Rank	Category 0	Category 1	Category 2	Category 3	Category 4
1	認めません (Not found) 1.44	陳旧性 (Old) 1.85	縮小 (Shrink) 1.18	増大 (Enlarge) 1.53	出現 (Emergence) 1.38
2	日付 (Date) 0.83	##萎縮 (##atrophy) 1.28	大 (In-size) 0.95	増強 (Intensify) 1.48	圧排 (Exclusion) 0.93
3	異常 (Abnormality) 0.78	萎縮 (Atrophy) 1.23	内側 (Inside) 0.88	拡大 (Expand) 1.04	おり (And) 0.93
4	内 (Inside) 0.78	脳萎縮 (Brain atrophy) 1.00	左視床 (Left thalamus) 0.87	術後 (Post-surgery) 1.00	後頭葉 (Occipital lobe) 0.86
5	明らか (Obvious) 0.77	石灰化 (Calcification) 0.92	コイル塞栓 (Coil embolization) 0.85	より (Compared with) 0.83	可能性 (Possibility) 0.84

#### Results of LIG in BiLSTM and BERT models

**Fig. 7:**
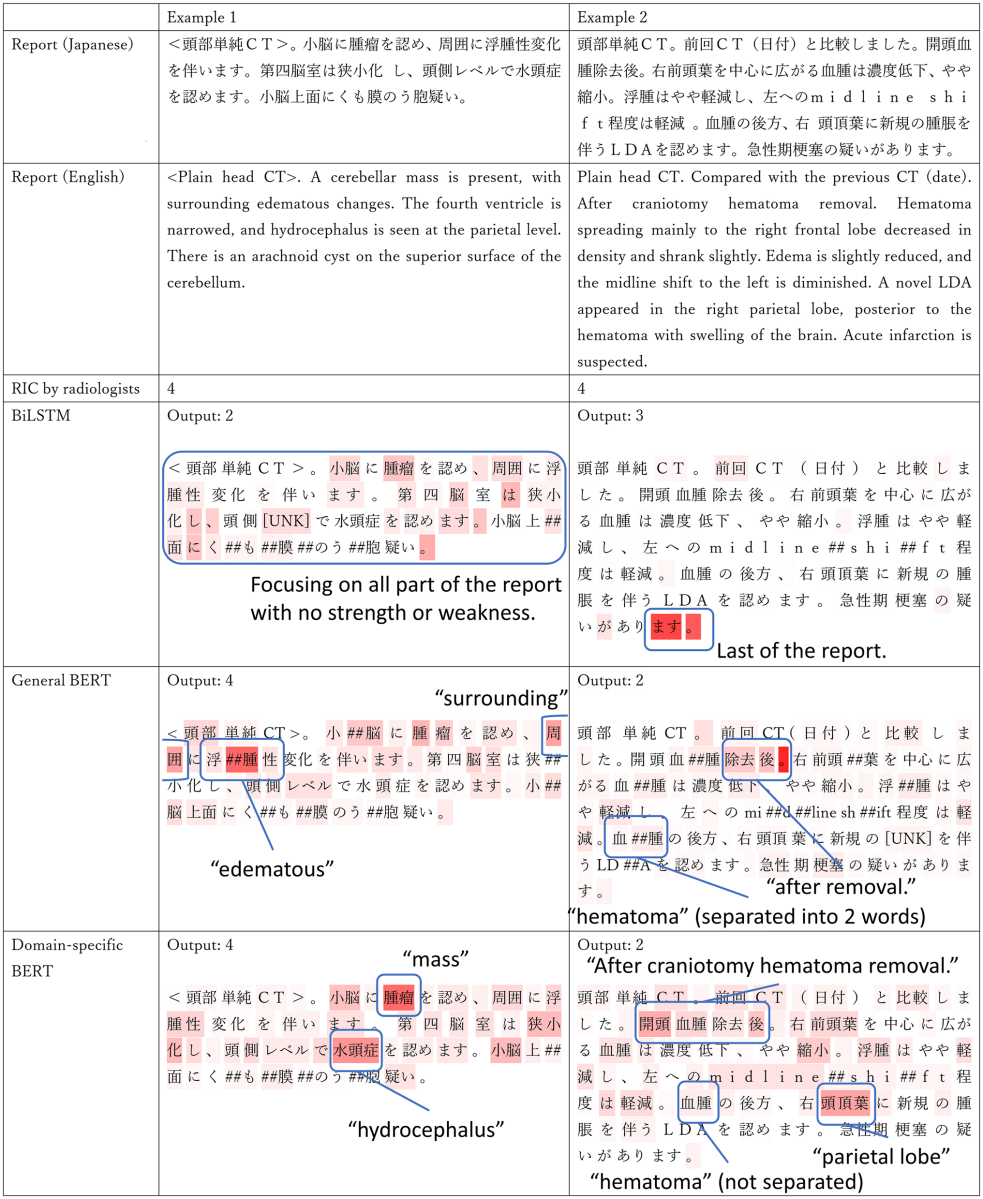
LIG results of two report examples by BiLSTM and BERT models. *LIG* layer integrated gradientsy, *BiLSTM* Bi-directional long–short-term memory, *BERT* bidirectional encoder representations from transformers

Figure 7 shows the LIG results of two report examples from the BiLSTM and BERT models. Example 1 contains the description of a mass and hydrocephalus, while Example 2 is a follow-up after craniotomy hematoma removal, which is a natural course, but an acute infarction is also suspected. BiLSTM focused on almost all tokens with no strength or weakness in Example 1, whereas only the end of the report in Example 2. On the other hand, BERT models tended to focus on explicit disease names, such as mass and hydrocephalus, and the focus by domain-specific BERT was even more accurate. However, even domain-specific BERT failed to recognize acute infarction and was unable to accurately categorize Example 2. In addition, it is noteworthy that unlike general BERT, which could not recognize medical terms, such as "hematoma" (separated into two words), domain-specific BERT recognized the word as it was.

## Discussion

Accurate assessment of the importance of radiology reports is awaited in terms of medical safety. In this study, we defined criteria for classifying the importance of reports, called RIC, compared the performance of NLP models for classifying RIC, and found that domain-specific BERT outperformed other models with the accuracy of 0.8434 ± 0.0063 and macro-average AUC of 0.9693 ± 0.0032.

Unlike statistical models, such as logistic regression, which only analyzes the frequency of words in reports, deep learning-based models (BiLSTM and BERT), are able to consider the order of each word. However, the superiority of BiLSTM over logistic regression was not observed, presumably because the radiology reports were too long to be included in the model with limited memory capacity. On the other hand, since the analysis of seemingly distant word-to-word engagements is crucial in NLP, the BERT models have overcome this problem using the multi-head attention layers. In this research, even between BERT models, the domain-specific BERT outperformed the general BERT. For one thing, the models have dictionaries of words that they can process, and how many medical terms are included is directly related to the model’s ability to process medical documents. In fact, as shown in Fig. 7, the word “hematoma” was split into two subwords in the general BERT, whereas the domain-specific BERT interpreted this word as it was. In addition, domain-specific models can specialize in interpreting phrases that are characteristic of medical documents.

Automatic classification of RIC is challenging in that reports of the same disease can be categorized as 2, 3, and 4 based on the history of the disease, and thus accurate assessment requires in-depth interpretation of the reports. This is typically illustrated by the fact that words related to disease names do not appear in the top five weights in logistic regression (Table 4), except for minor findings (which are often categorized as 1). In addition, multiple findings may occur, requiring a determination of which are the most important. Moreover, RIC assessment sometimes requires medical knowledge, such as the clinical natural history of disease. The fact that the disagreement rate of 14.3% between even neuroradiologists illustrates the difficulty of accurate assessment of reports. Thus, assists by systems using NLP can be helpful for radiologists.

In this study, scores for categories 3 and 4 tended to be lower than those for categories 0–2, and these higher category reports were often misclassified as category 2. Categories 3 and 4 were the minority, while category 2 was the majority, and this imbalanced data may have caused this result. In addition, category 3 and 4 reports tend to be longer, which means the reports contain more information, making it difficult for the models to accurately analyze higher category data. In addition, there are less common but important diseases (e.g., “annular axis subluxation” was found in a category 4 report in the test data set), so variations in findings between reports can occur, especially in higher categories. Thus, more train data are needed to overcome this variation problem. A previous study pointed out that the domain-specific BERT model can acquire medical knowledge through the pretraining process, and the train data set used in fine-tuning does not need to cover all variations [22], suggesting that the analogy by domain-specific BERT can work. However, it seems that this analogy did not work in this study, probably because of the small data set size and the complexity of RIC.

The superiority of BERT models over other models in extracting radiology reports with important findings has been reported previously. Yuta Nakamura, et al. reported the AUROC of the general BERT model in detecting actionable comments to be 0.9516 [11], which was slightly higher than the AUROC for category 4 in our study (0.9185 ± 0.0147 in general BERT), presumably because some reports in [11] contained explicitly contained word “actionable”, whereas there were no such reports in this study. Regarding the advantages of domain-specific BERT models, Imon Banerjee, et al. re-fine-tuned a domain-specific BERT (ClinicalBERT) using radiology reports and reported the benefits of re-fine-tuning in classifying reports with critical findings [23]. The narrower the domain in which BERT has specificity, the better the performance in that domain may be, and therefore, the development of a general BERT model domain-specific to radiology reports may improve the performance in this study.

This study has several limitations. First, this study was conducted at one institution; thus, the results cannot be generalized due to the limited number and variation of reports. The fact that the performance of the models for the validation and test data sets tended to decrease compared to the train data set implies that overfitting may have occurred, and using more reports collected from multiple institutions can overcome this issue. Second, although we aimed to detect important findings (RIC 4), such reports are rare in clinical practice, and the imbalance of data appeared. Third, since we had declared to the Ethics Review Board that we do not collect patient ids or names, the train and validation data and the test data may contain reports derived from the same patient. However, we consider the influence of this to be limited compared to the bias caused by the fact that the number of radiologists generating reports is limited due to the one-institution research.

We consider that this study needs to be developed in the future. For example, collecting reports from multiple facilities is desirable, as there is limited variation in the reports that can be obtained from one facility. Fortunately, Japan has a database of radiological images and reports called Japan Medical Image Database (J-MID) [24], which will help similar research in the near future.

In conclusion, we have established the five-point scale to evaluate the importance of radiology reports and demonstrated the superiority of the domain-specific BERT model. We hope for the accumulation of further studies analyzing other information at the same time, such as considering clinicians' order comments, patients' backgrounds, and, if possible, associated radiological images. These certainly have the potential to improve medical safety.

### Supplementary Information

Below is the link to the electronic supplementary material.

Supplementary file1 (DOCX 38 KB)

Supplementary file2 (DOCX 17 KB)

Supplementary file3 (DOCX 34 KB)

## Abbreviations

AUC: Area under the ROC curve
BERT: Bidirectional encoder representations from transformers
BiLSTM: Bidirectional long–short-term memory
CT: Computed tomography
LIG: Layer integrated gradients
NLP: Natural language processing
RIC: Report Importance Category
ROC: Receiver operating characteristic
