# Supplementary material for: Comparison of natural language processing algorithms in assessing the importance of head computed tomography reports written in Japanese
Source: Jpn J Radiol. 2024 Mar 29;42(7):697–708. doi: 10.1007/s11604-024-01549-9 (PMC11217108; doi:10.1007/s11604-024-01549-9)
Supplement: Supplementary file 1 — Supplementary file1 (DOCX 38 KB) [file 11604_2024_1549_MOESM1_ESM.docx]

(Supplementary material 1) Definitions of *Report Importance Category* (RIC)

Basics

- Classify head CT reports into the following five categories:

| RIC | Meaning | Explanation |
| --- | --- | --- |
| Category 0 | No findings | No findings are described. |
| Category 1 | Minor findings | Findings are described, but they do not require follow-up. |
| Category 2 | Routine follow-up | Findings are described and require "routine follow-up," meaning that clinically scheduled follow-up intervals do not need to be shortened because of the findings. |
| Category 3 | Careful follow-up | Findings are described and require "careful follow-up," meaning that the follow-up intervals for the findings should be shortened. |
| Category 4 | Examination or therapy | Novel or urgent findings are described, and further examination or therapy should be considered. |

- Classify reports based on "How much new (i.e., unknown) information is in the report for clinicians?" rather than "How severely ill is the patient? Whether the information is new or not depends on the impression of the report.
  - Example 1: “A mass is found in the skull.”
    - The clinician may not know the existence of the mass. -> This is a new finding.
  - Example 2: “A mass which was pointed out by the MRI examination is found in the skull.”
    - The clinician knows the existence of the mass. -> This is not a new finding.
- “Finding” refers to anything noted in the report, including intracranial/extracranial and normal variants.
- When more than one “finding” is described in the report, the category with the highest “finding” should be the category of the entire report. In this case, other “findings” should be ignored.
- When more than one differential diagnosis is described for a “finding” the category should be judged in the following order: (i) the category with the highest differential ranking; (ii) the highest category among the differential diseases when the differential rankings are equal.
- The category of the “finding” should be considered based on the severity of the “finding” itself and the changes over time.
- When only “findings” readable from the images are described and there is no description of the interpretation (e.g. differential diagnosis) and it is difficult to determine the category, the lowest possible grade classification based on the findings should be adopted.
- When a comparison with the previous examination is made in the report, the date of the previous examination (usually masked in this research) should not be considered. However, when the range from the previous examination is explicitly described in the report, it can be taken into account.
- When the specific clinical condition remains unclear in the report, the category should be “no findings”. (e.g. “The brain condition is the same as at the previous examination.”)
- When the description of changes in a “finding” is missing in the report, the “finding” should be considered unchanged.

Exact definition for each category

- Category 0: “No findings”
  - Reports without descriptions for any findings.
- Category 1: “Minor findings”
  - Reports with only minor “findings” that do not require CT follow-up.
    - Examples: chronic ischemic change, old infarction, brain atrophy, old hemorrhage, arachnoid cyst, calcification of basal ganglia, subcutaneous hemorrhage, sinus fluid, mastoid cell fluid, small meningioma, lipoma, old postoperative change (details are unknown), etc.
  - However, when the “findings” in this category have worsened from the previous time, categorize as 1 if follow-up is not necessary, and 2 if it is. In addition, categorize into 2 if there is a description of a specific disease that may require therapeutic intervention in the future (e.g., Alzheimer’s disease for brain atrophy).
- Category 2: “Routine follow-up”
  - Reports that correspond to any of the following:
    - (i) Require “routine follow-up”
      - “Routine follow-up” means that the intervals of follow-up that could be clinically scheduled do not need to be shortened for the findings.
      - The intervals of the “routine follow-up” may vary depending on the “findings”. (e.g. a few days for cerebral hemorrhage, and a few months for benign brain tumors)
      - Mainly refers to already known “findings” that have not worsened from the previous time or with only changes accountable due to the natural course.
    - (ii) The examination is intended for pre-treatment screening and the “finding” does not affect the treatment plan.
    - (iii) Minor “findings” and follow-up is recommended. (See the explanation of category 1.)
    - (iv) Follow-up is recommended for diagnosis, including when the existence of “findings” is unclear for such reasons as artifacts.
    - Note: “Findings” that are difficult to categorize (e.g. cervical spondylosis) should be categorized as 2, but may be changed to 1 or 3 depending on the severity.
    - Note: Reports that refer to follow-up after surgery and no changes should be categorized in 2, even if the "findings" have disappeared due to surgery.
- Category 3: “Careful follow-up”
  - Reports that correspond to any of the following:
    - (i) “Findings” have worsened except for minor ones.
    - (ii) Some changes appeared in the "finding", which is not natural, but can generally appear in the clinical course. (e.g. hemorrhage within the acute infarct lesion)
- Category 4: “Examination or therapy”
  - Reports that correspond to any of the following:
    - (i) Novel "findings" that may be the target of further investigation or treatment.
    - (ii) Some emergent "findings" that should be brought to the immediate attention of clinicians.

Detailed interpretations & definitions for each disease

- Post-surgery

| Example | Interpretation | RIC |
| --- | --- | --- |
| Old post-surgery change | Not recent and unknown surgery | 1 |
| Follow up after surgery | If there are no descriptions on interval change | 2 |
| (First time after the surgery)  Fluid collection or minor hemorrhage | Natural course as post-surgery | 2 |
| (First time after the surgery for brain tumors)  Remaining tumor | If the complete resection is clinically expected  (The existence of remaining tumor is not natural course) | 3 |
|  | If the existence of remaining tumor is clinically highly probable  (e.g. after biopsy) | 2 |
| (Not first time after the surgery)  Fluid collection or hemorrhage enlarges | Fluid collection or hemorrhage are worsened | 3 |
| (Not first time after the surgery)  Postoperative cavity enlarges | If swelling of brain diminishes | 2 |
|  | If fluid collection enlarges or cysts in tumors enlarges | 3 |

- Tumors/Masses

| Example | Interpretation | RIC |
| --- | --- | --- |
| Calcification in the cerebral falx, meningioma is suspected | Especially when the lesion is small | 1 |
| A mass exists, which was detected by the MRI scan | No description of enlargement/shrinkage, but the mass is already known | 2 |
| No interval change/ The tumor shrinks |  | 2 |
| Tumor enlarges |  | 3 |
| Post radiation therapy and low-density area (LDA) appeared around the mass | LDA can be due to (1) swelling of mass (RIC: 3), or (2) radiation therapy (RIC: 2). Both are possible. | 3 |
| Post radiation therapy and low-density area (LDA) appeared around the mass. MRI can be considered. | Further examination is expected. | 4 |
| Hemorrhage in the mass | Masses can be clinically complicated by hemorrhage, but not always | 3 |
| A novel mass (except minor findings) | Requires examination or therapy | 4 |
| Increased metastatic tumors in patients with already-known brain metastases | “Brain metastasis” is regarded one lesion in the entire brain | 3 |
| Enlarged ventricles suspected of dissemination in patients with known brain metastases | “Brain metastasis” and “dissemination” are regarded as different findings | 4 |
| The mass enlarges and midline shift appears | Midline shift is regarded as an expression of the degree of enlargement and is not considered as a finding by itself | 3 |

- Hydrocephalus

Note: When "the presence of enlarged ventricles is indicated " and "the cause is not specified," the patient is treated as if he/she has hydrocephalus.

| Example | Interpretation | RIC |
| --- | --- | --- |
| No interval changes in the ventricle size  (No mention of hydrocephalus) | Unclear whether there is ventricular enlargement | 0 |
| No interval changes in the ventricle enlargement | Ventricle are enlarged and the cause is not clarified  (Based on the “Note”) | 2 |
| Ventricle size slightly larger than last time | If the enlargement can be considered significant | 3 |
|  | If the enlargement can’t be considered significant | 2 |
| (In patient with shunt) Subdural hematoma present, suspected low CSF syndrome | When the hematoma can be considered as a new finding | 4 |
|  | When the shunt pressure adjustment treatment is necessary | 4 |
| Brain atrophy/ sulcus expansion | When there is no suggestion of diseases like “normal pressure hydrocephalus” | 1 |

- Brain infarction

| Example | Interpretation | RIC |
| --- | --- | --- |
| Chronic ischemic change/ old infarction |  | 1 |
| Old infarction increases | When follow-up is required | 2 |
| Acute infarction appeared |  | 4 |
| In the clinical course of acute infarction and surrounding LDA enlarges | Worsening of acute infarction | 3 |
| In the clinical course of acute infarction and LDA inside the lesion is clarified | Natural as the clinical course of acute infarction | 2 |
| Worsening of brain swelling | Brain swelling cannot be found in every patient | 3 |
| Hemorrhage in the lesion | Infarction can be clinically complicated by hemorrhage, but not always | 3 |
|  | When the mess effect appeared which can be urgent | 4 |
| “LDA is found” | LDA itself can be found in old infarction and masses, acute infarction, etc... The lowest RIC is considered | 1 |

- Hemorrhage

| Example | Interpretation | RIC |
| --- | --- | --- |
| (Except multiple trauma)  Hemorrhage in another part of the body" in a case with existing hemorrhage |  | 4 |
| Hematoma increases and extends to other parts | Regarded as the increase in hematoma | 3 |
| Hematoma volume remains unchanged |  | 2 |
| Subarachnoid hemorrhage (SAH) in a multiple trauma patient with already known cerebral hemorrhage | Can occur in the course of multiple trauma  (In this case, hemorrhagic changes are assembled and regarded as one lesion) | 3 |
| The appearance of infarcted foci associated with vasoconstriction after SAH | Regarded as an urgent finding | 4 |
| Suspected “fluid collection in the subarachnoid space” or “subdural hydrocele” | Highest RIC is selected for multiple differential diagnoses (“fluid collection in the subarachnoid space”: RIS 1 vs “subdural hydrocele”: RIS 2) | 2 |
|  | When the subdural hydrocele is a novel lesion | 3 |

- Sinusitis

| Example | Interpretation | RIC |
| --- | --- | --- |
| Fluid collection in the sinus cavity |  | 1 |
| Fluid collection increases | When there is no recommendation for follow-up | 1 |
|  | When there is recommendation for follow-up | 2 |
| Description such as "dental maxillary sinusitis" or "fungal sinusitis" |  | 2 |
| Sinusitis with bone fracture | High urgency | 4 |

Examples using actual reports

- Case 1

Head CT: Partially difficult to evaluate due to artifacts _(Description of the image quality, not particular findings->0)._ No obvious intracranial hemorrhage. No findings suggestive of acute infarction.

-> RIC: 0

- Case 2

Brain CT: Suspicion of a small, old infarction in the left cerebellum _(old infarction->1)_. No other intracranial abnormalities are found.

-> RIC: 1

- Case 3

Plain brain CT: LDA with swelling is seen in bilateral ACA regions, left MCA region, and left occipital lobe. Acute infarction course. The extent of infarction is generally similar to the previous MRI (Date). No obvious hemorrhagic complications. _(Cerebral infarction course, no marked difference from MRI->2)_ Strong focal atrophy is observed in the medial part of bilateral temporal lobes. _(Alzheimer's disease is suggested->2)_

-> RIC: 2

- Case 4

<Plain head CT>. Compared with the previous CT (Date). The CSF space between the bilateral skull and brain parenchyma is enlarged. The cerebral sulcus has narrowed. There is no obvious hyper density area. Bilateral subdural fluid collection is suspected. _(Appearance of fluid collection or enlargement of existing one->3)_ No other new intracranial lesions. Small soft shadow is found in the left maxillary sinus. Suspected sinusitis. _(Minor_ _finding->1)_

-> RIC: 3

- Case 4

<Plain head CT>. Compared with CT (Date). High density shadow suspected of hemorrhage in the right thalamus has decreased in the density compared to the previous time and is in the process of absorption. _(Hemorrhage process, improving->2)_ No obvious new hemorrhage. Low density shadow suggesting edematous change around the hematoma. Slightly enlarged since last time. _(LDA worsening→3)_ Enlargement of bilateral ventricles, enlargement of sylvian fissure, and narrowing of the sulcus of the higher cranial part of the brain. Same as last time. _(Enlargement of ventricles of unknown cause (probably normal pressure hydrocephalus), no significant change from the previous CT->2)_ Fluid collection is found in ethmoid sinus and sphenoid sinus. _(Minor finding->1)_

->RIC: 3

- Case 5

<Plain head CT>

A right subdural hematoma is observed. Internal areas with high density can be found. Narrowing of the cerebral sulcus is seen on the parietal side. _(New hemorrhage->4)_ No midline shift is present. No infarction is suspected.

->RIC: 4
