# Supplementary material for: Comparison of natural language processing algorithms in assessing the importance of head computed tomography reports written in Japanese
Source: Jpn J Radiol. 2024 Mar 29;42(7):697–708. doi: 10.1007/s11604-024-01549-9 (PMC11217108; doi:10.1007/s11604-024-01549-9)
Supplement: Supplementary file 2 — Supplementary file2 (DOCX 17 KB) [file 11604_2024_1549_MOESM2_ESM.docx]

(Supplementary material 2) Hyperparameters in BiLSTM and BERT models decided using Optuna

|  | BiLSTM | General BERT | Domain-specific BERT |
| --- | --- | --- | --- |
| Embedding dimensions | 400 | N/A | N/A |
| Hidden dimensions | 700 | N/A | N/A |
| Input shape for the final fully connected layer | 1400# | 768† | 768† |
| Learning rate | 2e-3 | 3e-5 | 2e-5 |
| Batch size | 128 | 4* | 4* |

Note:

#Since the dimension of the output vector for the forward and reverse directions is 700 (the same as the hidden dimensions), this is doubled.

†Defined by the pretrained model.

* For BERT models, the batch size was set to 4 due to GPU memory limitations.

BiLSTM was trained over100 epochs, while the BERT models were trained over 10 epochs because the BERT models were pre-trained models. The models with the lowest validation loss were used for the final evaluation.
