# Supplementary material for: Comparison of natural language processing algorithms in assessing the importance of head computed tomography reports written in Japanese
Source: Jpn J Radiol. 2024 Mar 29;42(7):697–708. doi: 10.1007/s11604-024-01549-9 (PMC11217108; doi:10.1007/s11604-024-01549-9)
Supplement: Supplementary file 3 — Supplementary file3 (DOCX 34 KB) [file 11604_2024_1549_MOESM3_ESM.docx]

(Supplementary material 3) Performance of each fold for the train, validation, and test dataset

Logistic Regression

| Fold | Fold 1 | | | Fold 2 | | | Fold 3 | | | Fold 4 | | | Fold 5 | | |
| --- | --- | --- | --- | --- | --- | --- | --- | --- | --- | --- | --- | --- | --- | --- | --- |
|  | Train | Validation | Test | Train | Validation | Test | Train | Validation | Test | Train | Validation | Test | Train | Validation | Test |
| Accuracy | 0.9916 | 0.7973 | 0.7842 | 0.9916 | 0.7705 | 0.7989 | 0.9895 | 0.8138 | 0.7828 | 0.9895 | 0.8138 | 0.7895 | 0.9916 | 0.7970 | 0.7802 |
| Macro F1 | 0.9911 | 0.6856 | 0.6886 | 0.9908 | 0.6785 | 0.7207 | 0.9909 | 0.7330 | 0.6793 | 0.9895 | 0.7396 | 0.6982 | 0.9919 | 0.7111 | 0.6840 |
| Macro AUC | 0.9999 | 0.9230 | 0.9139 | 0.9998 | 0.9344 | 0.9222 | 0.9998 | 0.9206 | 0.9190 | 0.9998 | 0.9334 | 0.9299 | 0.9998 | 0.9350 | 0.9164 |
| F1 for category 0 | 0.9972 | 0.9503 | 0.9427 | 0.9958 | 0.9457 | 0.9427 | 0.9986 | 0.9451 | 0.9422 | 0.9986 | 0.9405 | 0.9386 | 0.9986 | 0.9318 | 0.9507 |
| F1 for category 1 | 0.9859 | 0.8580 | 0.8418 | 0.9891 | 0.8139 | 0.8305 | 0.9835 | 0.8328 | 0.8193 | 0.9859 | 0.8328 | 0.8168 | 0.9866 | 0.8358 | 0.8184 |
| F1 for category 2 | 0.9938 | 0.8078 | 0.8012 | 0.9929 | 0.7903 | 0.8145 | 0.9896 | 0.8294 | 0.8060 | 0.9896 | 0.8359 | 0.8161 | 0.9924 | 0.8189 | 0.8024 |
| F1 for category 3 | 0.9918 | 0.3784 | 0.4386 | 0.9862 | 0.3095 | 0.5505 | 0.9862 | 0.5333 | 0.4954 | 0.9835 | 0.5750 | 0.4673 | 0.9918 | 0.4444 | 0.4912 |
| F1 for category 4 | 0.9867 | 0.4333 | 0.4186 | 0.9900 | 0.5333 | 0.4651 | 0.9967 | 0.5246 | 0.3333 | 0.9900 | 0.5135 | 0.4524 | 0.9900 | 0.5246 | 0.3571 |
| AUC for category 0 | 1.0000 | 0.9972 | 0.9951 | 1.0000 | 0.9968 | 0.9954 | 1.0000 | 0.9978 | 0.9953 | 1.0000 | 0.9970 | 0.9956 | 1.0000 | 0.9948 | 0.9970 |
| AUC for category 1 | 0.9998 | 0.9663 | 0.9535 | 0.9997 | 0.9425 | 0.9529 | 0.9997 | 0.9489 | 0.9514 | 0.9996 | 0.9562 | 0.9535 | 0.9996 | 0.9627 | 0.9526 |
| AUC for category 2 | 0.9998 | 0.8995 | 0.8983 | 0.9993 | 0.9011 | 0.9051 | 0.9992 | 0.9131 | 0.9026 | 0.9992 | 0.9189 | 0.9112 | 0.9993 | 0.9175 | 0.8990 |
| AUC for category 3 | 1.0000 | 0.8678 | 0.8948 | 0.9999 | 0.9039 | 0.8968 | 1.0000 | 0.8747 | 0.8879 | 1.0000 | 0.8916 | 0.9068 | 1.0000 | 0.8943 | 0.8721 |
| AUC for category 4 | 1.0000 | 0.8840 | 0.8279 | 1.0000 | 0.9277 | 0.8610 | 1.0000 | 0.8681 | 0.8577 | 1.0000 | 0.9035 | 0.8823 | 1.0000 | 0.9055 | 0.8611 |

Note: Since five-fold cross validation was performed, the train and validation dataset are different among folds.

BiLSTM

| Fold | Fold 1 | | | Fold 2 | | | Fold 3 | | | Fold 4 | | | Fold 5 | | |
| --- | --- | --- | --- | --- | --- | --- | --- | --- | --- | --- | --- | --- | --- | --- | --- |
|  | Train | Validation | Test | Train | Validation | Test | Train | Validation | Test | Train | Validation | Test | Train | Validation | Test |
| Accuracy | 0.9052 | 0.7605 | 0.7614 | 0.9480 | 0.7605 | 0.7788 | 0.8856 | 0.7869 | 0.7587 | 0.8730 | 0.7735 | 0.7721 | 0.8495 | 0.7836 | 0.7560 |
| Macro F1 | 0.8270 | 0.5942 | 0.5930 | 0.9159 | 0.6449 | 0.6540 | 0.7648 | 0.5944 | 0.5616 | 0.7307 | 0.5966 | 0.6001 | 0.7028 | 0.5871 | 0.5730 |
| Macro AUC | 0.9807 | 0.9128 | 0.9059 | 0.9925 | 0.9188 | 0.9247 | 0.9732 | 0.9215 | 0.9092 | 0.9727 | 0.9146 | 0.9077 | 0.9589 | 0.9037 | 0.9026 |
| F1 for category 0 | 0.9944 | 0.9162 | 0.9412 | 0.9944 | 0.9560 | 0.9643 | 0.9916 | 0.9605 | 0.9677 | 0.9874 | 0.9540 | 0.9581 | 0.9888 | 0.9655 | 0.9537 |
| F1 for category 1 | 0.9562 | 0.8148 | 0.8282 | 0.9736 | 0.7848 | 0.8370 | 0.9542 | 0.8307 | 0.8214 | 0.9476 | 0.8108 | 0.8426 | 0.9045 | 0.8343 | 0.8098 |
| F1 for category 2 | 0.9099 | 0.7930 | 0.7907 | 0.9524 | 0.7788 | 0.7890 | 0.8868 | 0.8202 | 0.7833 | 0.8776 | 0.7986 | 0.7913 | 0.8575 | 0.8076 | 0.7795 |
| F1 for category 3 | 0.4291 | 0.0345 | 0.1266 | 0.7857 | 0.2985 | 0.3095 | 0.1854 | 0.0800 | 0.0328 | 0.1224 | 0.0000 | 0.0645 | 0.0632 | 0.0426 | 0.0000 |
| F1 for category 4 | 0.8456 | 0.4127 | 0.2785 | 0.8732 | 0.4063 | 0.3704 | 0.8059 | 0.2807 | 0.2025 | 0.7185 | 0.4194 | 0.3441 | 0.7000 | 0.2857 | 0.3218 |
| AUC for category 0 | 0.9995 | 0.9970 | 0.9904 | 1.0000 | 0.9961 | 0.9913 | 0.9997 | 0.9985 | 0.9886 | 0.9999 | 0.9955 | 0.9949 | 0.9996 | 0.9925 | 0.9881 |
| AUC for category 1 | 0.9945 | 0.9524 | 0.9561 | 0.9985 | 0.9392 | 0.9575 | 0.9950 | 0.9525 | 0.9589 | 0.9916 | 0.9380 | 0.9625 | 0.9858 | 0.9618 | 0.9501 |
| AUC for category 2 | 0.9770 | 0.8794 | 0.8872 | 0.9925 | 0.8854 | 0.9000 | 0.9671 | 0.9239 | 0.8769 | 0.9613 | 0.9013 | 0.8906 | 0.9459 | 0.8921 | 0.8720 |
| AUC for category 3 | 0.9493 | 0.8406 | 0.8420 | 0.9841 | 0.8808 | 0.8846 | 0.9234 | 0.8582 | 0.8512 | 0.9392 | 0.8603 | 0.8342 | 0.9006 | 0.8261 | 0.8339 |
| AUC for category 4 | 0.9833 | 0.8947 | 0.8535 | 0.9876 | 0.8927 | 0.8903 | 0.9811 | 0.8744 | 0.8704 | 0.9713 | 0.8780 | 0.8564 | 0.9627 | 0.8458 | 0.8689 |

Note: Since five-fold cross validation was performed, the train and validation dataset are different among folds.

General BERT

| Fold | Fold 1 | | | Fold 2 | | | Fold 3 | | | Fold 4 | | | Fold 5 | | |
| --- | --- | --- | --- | --- | --- | --- | --- | --- | --- | --- | --- | --- | --- | --- | --- |
|  | Train | Validation | Test | Train | Validation | Test | Train | Validation | Test | Train | Validation | Test | Train | Validation | Test |
| Accuracy | 0.8738 | 0.8208 | 0.8097 | 0.9597 | 0.8090 | 0.8083 | 0.9790 | 0.8305 | 0.8432 | 0.9355 | 0.8255 | 0.8244 | 0.8923 | 0.8205 | 0.7962 |
| Macro F1 | 0.8119 | 0.7349 | 0.7109 | 0.9403 | 0.7847 | 0.7448 | 0.9721 | 0.7556 | 0.7641 | 0.9106 | 0.7320 | 0.7352 | 0.8421 | 0.7546 | 0.6885 |
| Macro AUC | 0.9695 | 0.9532 | 0.9423 | 0.9981 | 0.9602 | 0.9517 | 0.9989 | 0.9575 | 0.9590 | 0.9948 | 0.9630 | 0.9572 | 0.9861 | 0.9495 | 0.9459 |
| F1 for category 0 | 0.9860 | 0.9773 | 0.9772 | 0.9944 | 0.9721 | 0.9725 | 0.9958 | 0.9718 | 0.9821 | 0.9944 | 0.9659 | 0.9774 | 0.9958 | 0.9600 | 0.9818 |
| F1 for category 1 | 0.8894 | 0.8452 | 0.8406 | 0.9805 | 0.8146 | 0.8471 | 0.9827 | 0.8391 | 0.8731 | 0.9636 | 0.8701 | 0.8678 | 0.9042 | 0.8470 | 0.8489 |
| F1 for category 2 | 0.8889 | 0.8315 | 0.8270 | 0.9636 | 0.8024 | 0.8161 | 0.9802 | 0.8478 | 0.8555 | 0.9352 | 0.8450 | 0.8361 | 0.9069 | 0.8306 | 0.8107 |
| F1 for category 3 | 0.6992 | 0.5301 | 0.5253 | 0.8502 | 0.6496 | 0.6056 | 0.9479 | 0.6364 | 0.5905 | 0.8038 | 0.6018 | 0.6176 | 0.6936 | 0.5676 | 0.3750 |
| F1 for category 4 | 0.5959 | 0.4906 | 0.3846 | 0.9128 | 0.6849 | 0.4828 | 0.9536 | 0.4828 | 0.5195 | 0.8561 | 0.3774 | 0.3768 | 0.7101 | 0.5679 | 0.4259 |
| AUC for category 0 | 0.9996 | 0.9942 | 0.9955 | 1.0000 | 0.9949 | 0.9914 | 1.0000 | 0.9996 | 0.9942 | 1.0000 | 0.9997 | 0.9996 | 1.0000 | 0.9897 | 0.9992 |
| AUC for category 1 | 0.9826 | 0.9718 | 0.9659 | 0.9987 | 0.9449 | 0.9665 | 0.9996 | 0.9725 | 0.9721 | 0.9977 | 0.9783 | 0.9754 | 0.9933 | 0.9771 | 0.9757 |
| AUC for category 2 | 0.9607 | 0.9231 | 0.9209 | 0.9975 | 0.9253 | 0.9300 | 0.9982 | 0.9497 | 0.9437 | 0.9891 | 0.9536 | 0.9327 | 0.9795 | 0.9384 | 0.9244 |
| AUC for category 3 | 0.9652 | 0.9209 | 0.9274 | 0.9957 | 0.9641 | 0.9493 | 0.9978 | 0.9379 | 0.9466 | 0.9926 | 0.9412 | 0.9491 | 0.9824 | 0.9384 | 0.9286 |
| AUC for category 4 | 0.9394 | 0.9558 | 0.9015 | 0.9984 | 0.9717 | 0.9214 | 0.9991 | 0.9276 | 0.9382 | 0.9946 | 0.9420 | 0.9293 | 0.9756 | 0.9036 | 0.9019 |

Note: Since five-fold cross validation was performed, the train and validation dataset are different among folds.

Domain-specific BERT

| Fold | Fold 1 | | | Fold 2 | | | Fold 3 | | | Fold 4 | | | Fold 5 | | |
| --- | --- | --- | --- | --- | --- | --- | --- | --- | --- | --- | --- | --- | --- | --- | --- |
|  | Train | Validation | Test | Train | Validation | Test | Train | Validation | Test | Train | Validation | Test | Train | Validation | Test |
| Accuracy | 0.9635 | 0.8425 | 0.8405 | 0.9744 | 0.8442 | 0.8539 | 0.9581 | 0.8540 | 0.8418 | 0.9593 | 0.8708 | 0.8458 | 0.9107 | 0.8523 | 0.8351 |
| Macro F1 | 0.9409 | 0.8079 | 0.7787 | 0.9655 | 0.8168 | 0.8173 | 0.9389 | 0.7993 | 0.7879 | 0.9488 | 0.8123 | 0.7874 | 0.8699 | 0.7748 | 0.7419 |
| Macro AUC | 0.9969 | 0.9711 | 0.9680 | 0.9990 | 0.9648 | 0.9684 | 0.9975 | 0.9698 | 0.9722 | 0.9973 | 0.9725 | 0.9733 | 0.9881 | 0.9645 | 0.9645 |
| F1 for category 0 | 0.9972 | 0.9432 | 0.9772 | 0.9958 | 0.9889 | 0.9864 | 0.9945 | 0.9778 | 0.9776 | 0.9958 | 0.9724 | 0.9774 | 0.9888 | 0.9714 | 0.9818 |
| F1 for category 1 | 0.9759 | 0.8825 | 0.8706 | 0.9815 | 0.8483 | 0.8873 | 0.9663 | 0.8667 | 0.8668 | 0.9619 | 0.8839 | 0.8747 | 0.9298 | 0.9032 | 0.8825 |
| F1 for category 2 | 0.9718 | 0.8399 | 0.8541 | 0.9758 | 0.8405 | 0.8449 | 0.9647 | 0.8697 | 0.8529 | 0.9610 | 0.8994 | 0.8563 | 0.9183 | 0.8545 | 0.8454 |
| F1 for category 3 | 0.8724 | 0.6355 | 0.6202 | 0.9091 | 0.6596 | 0.6949 | 0.8792 | 0.6735 | 0.6515 | 0.8937 | 0.6809 | 0.6346 | 0.7222 | 0.5479 | 0.4545 |
| F1 for category 4 | 0.8873 | 0.7385 | 0.5714 | 0.9651 | 0.7470 | 0.6731 | 0.8897 | 0.6087 | 0.5909 | 0.9313 | 0.6250 | 0.5941 | 0.7905 | 0.5970 | 0.5455 |
| AUC for category 0 | 1.0000 | 0.9965 | 0.9959 | 1.0000 | 0.9993 | 0.9973 | 1.0000 | 0.9996 | 0.9990 | 1.0000 | 0.9994 | 0.9957 | 0.9998 | 0.9991 | 0.9990 |
| AUC for category 1 | 0.9984 | 0.9750 | 0.9796 | 0.9995 | 0.9689 | 0.9819 | 0.9988 | 0.9786 | 0.9830 | 0.9977 | 0.9778 | 0.9812 | 0.9918 | 0.9844 | 0.9807 |
| AUC for category 2 | 0.9967 | 0.9357 | 0.9506 | 0.9987 | 0.9398 | 0.9487 | 0.9962 | 0.9599 | 0.9538 | 0.9939 | 0.9685 | 0.9520 | 0.9795 | 0.9471 | 0.9409 |
| AUC for category 3 | 0.9928 | 0.9643 | 0.9631 | 0.9970 | 0.9456 | 0.9594 | 0.9956 | 0.9543 | 0.9655 | 0.9960 | 0.9570 | 0.9723 | 0.9811 | 0.9571 | 0.9582 |
| AUC for category 4 | 0.9967 | 0.9838 | 0.9510 | 0.9999 | 0.9702 | 0.9545 | 0.9968 | 0.9568 | 0.9597 | 0.9988 | 0.9601 | 0.9652 | 0.9881 | 0.9349 | 0.9434 |

Note: Since five-fold cross validation was performed, the train and validation dataset are different among folds.
